# Supplementary figures and images for: tACS entrains neural activity while somatosensory input is blocked
Source: PLoS Biol. 2020 Oct 1;18(10):e3000834. doi: 10.1371/journal.pbio.3000834 (PMC7553316; doi:10.1371/journal.pbio.3000834)

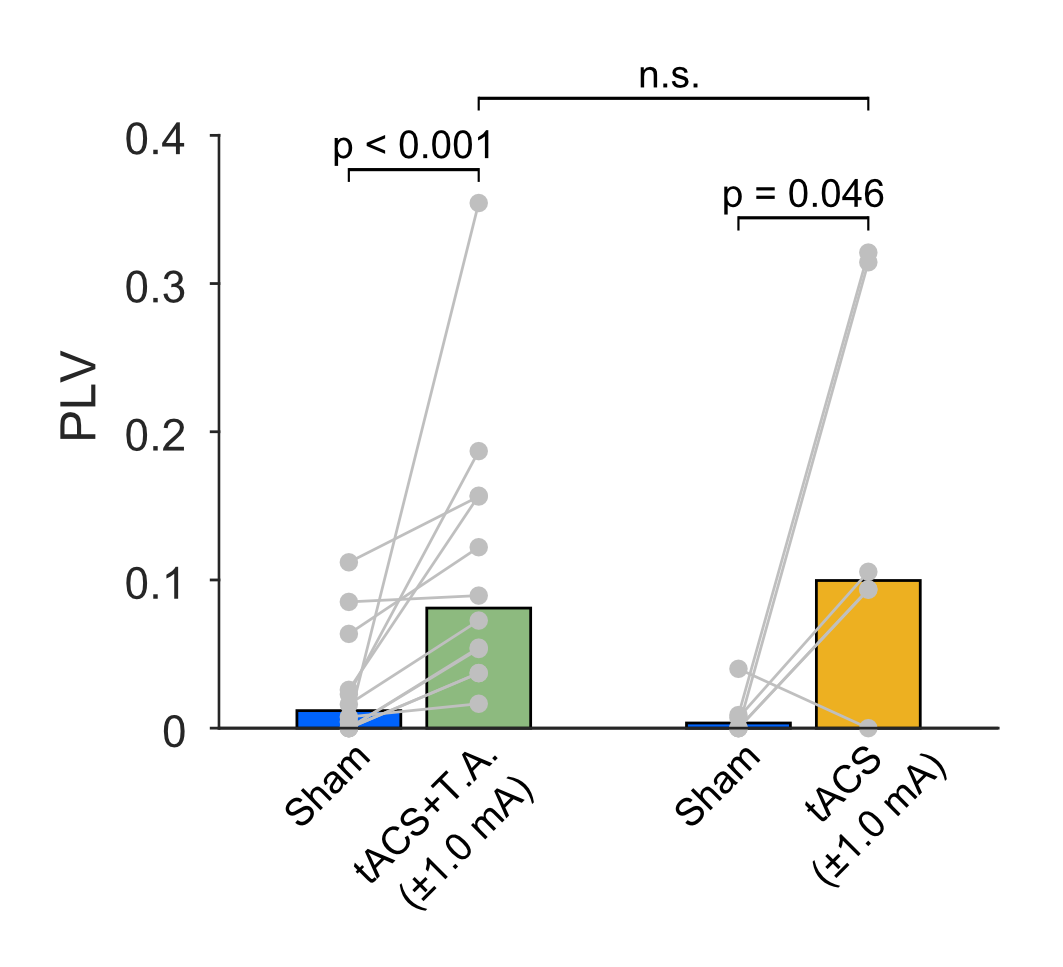

Supplement: S1 Fig — Data shown in the same style as Fig 3C and 3D. PLVs for individual neurons are indicated by gray points, with lines connecting observations from the same cell. As before, tACS increases neuronal entrainment during TA (green) and control sessions (yellow), compared to the corresponding sham conditions. However, no significant difference (n.s; p > 0.05) was detected between tACS and tACS + TA. See S3 Data for individual values of each data point. n.s, not significant; PLV, phase-locking value; TA, topical anesthesia; tACS, transcranial alternating current stimulation. (TIF) [file pbio.3000834.s001.tif]

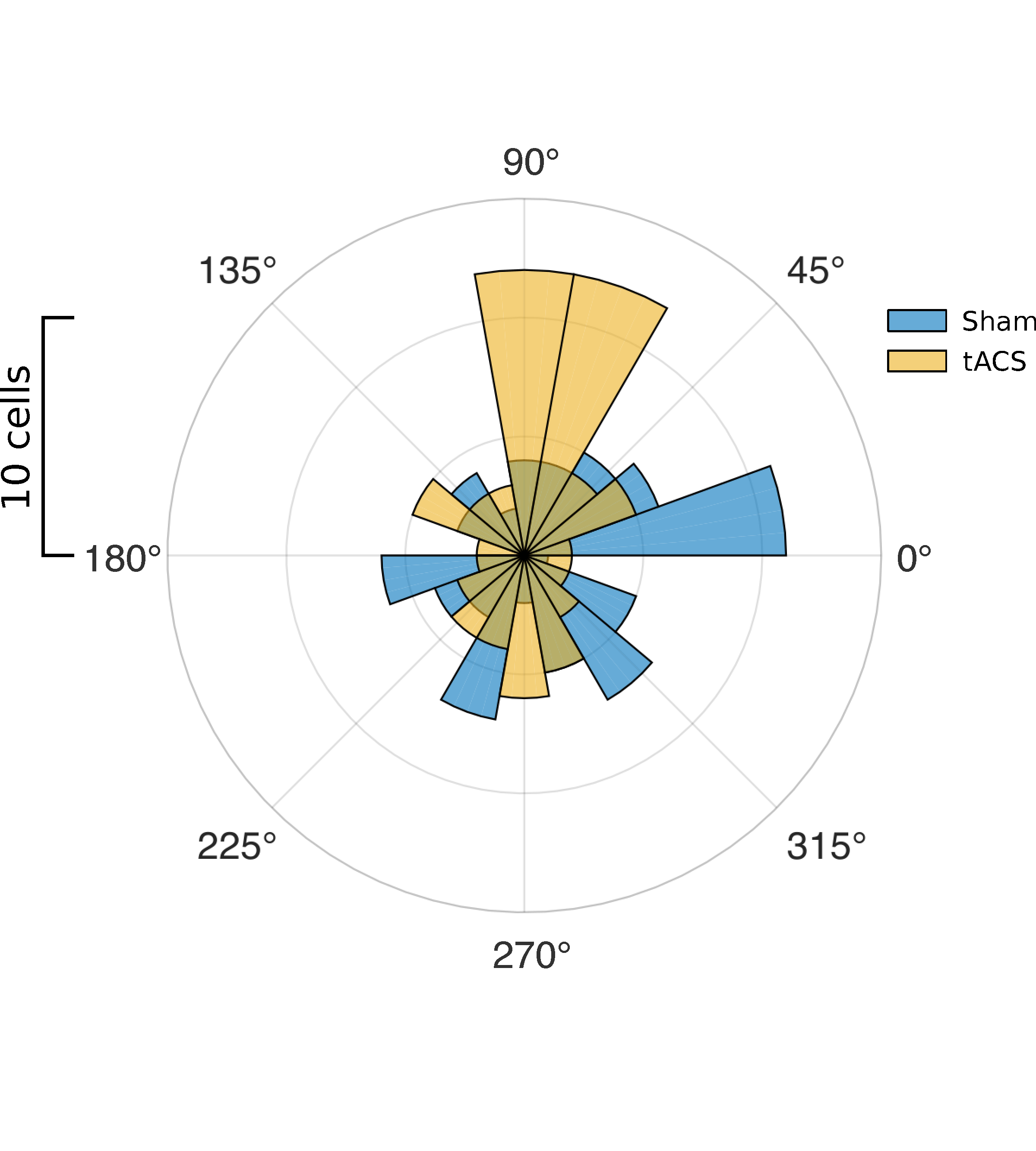

Supplement: S2 Fig — Distribution of preferred firing phases for the neurons shown in Fig 3 during sham (blue) and 20-Hz tACS (yellow) stimulation. During tACS, neurons preferentially fired near the peak of the tACS waveform (90°), but no such concentration was observed under sham conditions. Note that the preferred phase estimates for sham stimulation are noisy because neurons were minimally entrained to the 20-Hz component of the LFP (Fig 3). See S2 Data for individual values of each data point. LFP, local field potential; tACS, transcranial alternating current stimulation. (TIF) [file pbio.3000834.s002.tif]
