# Supplementary material for: tACS entrains neural activity while somatosensory input is blocked
Source: PLoS Biol. 2020 Oct 1;18(10):e3000834. doi: 10.1371/journal.pbio.3000834 (PMC7553316; doi:10.1371/journal.pbio.3000834)
Supplement: Attachment — Submitted filename: R2R (dummy file).docx [file pbio.3000834.s006.docx]

Blank R2R
