# Supplementary material for: tACS entrains neural activity while somatosensory input is blocked
Source: PLoS Biol. 2020 Oct 1;18(10):e3000834. doi: 10.1371/journal.pbio.3000834 (PMC7553316; doi:10.1371/journal.pbio.3000834)
Supplement: Attachment — Submitted filename: Reviewer Response.docx [file pbio.3000834.s007.docx]

We would like to thank all three Reviewers for their supportive and constructive comments on our manuscript. We have revised the manuscript to address the Reviewer’s concerns, especially about other possible indirect mechanisms, including retinal and cranial nerve stimulation. Detailed point-by-point responses to each comment are below, with the Reviewer’s comments in bold, our reply in roman, and quotes from the manuscript indented. Thank you again for your time and expertise.

Sincerely,

Pedro Vieira, Matthew R. Krause, and Christopher Pack

**Reviewer #1: This manuscript addresses the question whether physiological effects of tACS might be due to somatosensory electrical stimulation, and might be seen as a general reply to Asamoah et al. (2019). I have the following comments on the manuscript.** 
**1)  In the Transcranial Alternating Current Stimulation subsection of the methods part and referring to Huang et al. (2017), it is stated:
"For the V4 site, a montage consisting of Fp1/P7 was found to produce field of ~1 V/m with 1 mA of stimulating current, which approximates field strengths achievable in humans [21]." This is one of the several references to Huang et al. (2017), and in many of them the feasibility of reaching field strength of 1V/m is claimed. 
However, Huang et al. state in their discussion: "Electric field magnitudes in cortex can be as high as 0.2 V/m for 1 mA stimulation current. For typical electrode configurations used in clinical trials maximal field intensity can reach 0.4 V/m when applying 2 mA. More extended areas can reach a value of 0.16 V/m (95th percentile) under 2 mA stimulation. For some electrode montages, current is shunted into deeper areas through highly conductive CSF with maximal values reaching 0.12 V/m. Based on the present data, previous modeling estimates with maximal intensities around 1 V/m and 95th percentile of 0.35 V/m have to be downwards corrected by a factor of two." 
From this, I conclude that a field with a peak of about 1V/m requires application of tACS with amplitude of more than 4 mA, which is much bigger than usual tACS intensities. Please clarify this and make sure that the comparison with the achievable intensities in human studies is correct.**

The Reviewer appears to be quoting from the first version of the article published by Huang et al. (2017). In a subsequent correction (<https://elifesciences.org/articles/35178>), they reported that a technical error caused voltage and field strength measurements reported in the original paper to be halved. On page 12 of the corrected manuscript, Huang et al. now report:

Electric field magnitudes in cortex can be as high as 0.4 V/m for 1 mA stimulation current. For typical electrode configurations used in clinical trials maximal field intensity can reach 0.8 V/m when applying 2 mA.”

In the correction notice itself, Huang et al. also observe that:

In fact, with this correction there is now remarkable agreement of our results with existing models, as well as *in-vivo* measurements.

Jackson et al. (2016) also review estimates of field strength from computational models:

Though methodological approaches vary across groups, those modeling studies using realistic anatomy have converged in their estimates of peak electrical fields generated during tDCS to between 0.2–0.5 V/m (0.05–0.14 A/m2 current density) for a 1 mA intensity (Miranda, Lomarev, and Hallett (2006); Datta et al. (2009); Sadleir et al. (2010)). The electric field scales linearly with current intensity such that 2 mA would produce a range of 0.4–1 V/m.

The data in our Figure 3, collected with field strengths of 0.3 V/m (circles) and 1 V/m (diamonds), therefore bracket the best available estimates for field strengths achievable with two electrode-montages in humans. To further clarify this point, we have added references to Jackson et al. (2016) in the introduction and discussion.

**2)  Related to my previous point, fields produced in usual human tACS studies seem to be 25% of fields generated in the current study (for V4 experiment).**

Yes. Simulations by Alekseichuk et al. (2019) indicate that, when stimulation parameters are held constant, tACS will produce an electric field that is three times stronger in the head of a capuchin monkey than a human. Since macaques are somewhat larger than capuchins, the difference between humans and macaque monkeys may be slightly less. Nevertheless, we agree that passing ±1 mA between Fp1 and P7 will likely produce a field closer to 0.3 V/m than 1 V/m in humans.

However, as the studies referenced above indicate, field strengths of 1 V/m are nevertheless achievable in humans. In this particular example, increasing the stimulating current to ±2 mA (still within established safety limits) will double the field strength. The rest of the gap may be closed by improved electrode placement—many scalp locations are not accessible to us because of the animals’ implants. Moreover, the neural effects of a given field strength are constant across species, regardless of the stimulation amplitude needed to create that field. Indeed, the take-home message of Alekseichuk et al. (2019) is that tACS currents should be adjusted to produce translationally-relevant field strengths across animal models, just as we did here.

**How could PLV results of this paper be generalized to human studies?**

This is the million dollar question! Little is known about the role of spike timing in human behavior, since measuring it requires invasive recordings with high impedance electrodes. In one of the few available data sets, Rutishauser et al. (2010) reported that small increases in theta-band phase-locking* were associated with forgotten (0.015) vs. successfully-remembered (0.022) stimuli. These data are correlational rather than causal, but suggest the potential usefulness of methods that can induce small changes in spike timing, like tACS.

Much more data is available from the animal literature, which we reviewed in Krause et al. (2019):

Our data do not directly address this point, but the entrainment changes reported here, particularly during 20-Hz stimulation, are comparable to those linked with behavioral changes. Specifically, increases of ∼0.1 PLV have been associated with changes in sensory representations and working memory (Bahmani et al., 2018; van Wingerden et al., 2010b), perceptual organization (Vinck et al., 2010), behavioral state (Vinck et al., 2015), learning and reward expectancy (van Wingerden et al., 2010a), and the retrieval of memories (Foster et al., 2013). Changes in synchronous activity appear to underlie momentary lapses in performance (Rutishauser et al., 2010) and serious pathological conditions (Spencer et al., 2004), so even small changes in spike timing, when distributed across a network of neurons, may exert a powerful influence on behavior.

We have added a brief mention of these findings to the discussion on p. 7-8:

The resulting changes in spike-timing are similar to those reported in conjunction with therapeutically-relevant changes in human and animal behavior (reviewed in Krause et al., 2019), suggesting that non-sensory effects of tACS may provide an effective method for adjusting humans’ mental states.

* The values in Rutishauser et al. (2010) are reported as spike-field coherence. Under some simplifying assumptions, these are mathematically equivalent to the phase-locking values reported in our manuscript. See Vinck et al. (2012) for more about the relationship between spike-field coherence and phase-locking values.

**Moreover, could it be that the contribution of peripheral stimulation (compared to the contribution from direct stimulation) be more significant at lower tACS intensities?**

Both the peripheral and direct effects should scale with the stimulation amplitude, and therefore, the strength of the induced electric field. The polarization of a neuron’s membrane potential increases linearly with the field strength, as demonstrated by Deans, Powell, and Jefferys (2007) *in vitro* and Voroslakos et al. (2018) in a small animal model. A similar dose-response relationship has been shown for the neuron’s spiking output (Johnson et al., 2019).

We therefore agree that if peripheral stimulation played a role, its relative influence could be greater at lower stimulation intensities where the intracranial electric field is weaker. However, this would predict that the effects of topical anesthesia would increase as stimulation intensity decreased, which is not observed in our data. Indeed, we find little effect of somatosensory stimulation at any tACS intensity: there was no significant difference in entrainment when stimulation was applied with or without topical anesthesia and equivalence testing indicates that this is not due to a lack of statistical power (Figure 3). Our behavioral assay suggests that if there is residual somatosensory input, it must be relatively weak, especially in the 1 mA conditions (Figure 3, diamonds and Figure S1). Nevertheless, the possibility of a neural influence via other, indirect pathways is hard to formally exclude and they would naturally be strongest when the intracranial electric field is the weakest. We acknowledge this possibility in the conclusion on page 8:

The combined effects of sensory input, nerve fiber stimulation, and direct polarization will necessarily vary across stimulation protocols, targeted brain regions, and behavioral tasks, frustrating any simplistic attempt at interpretation.

**3)      Due to the electrode positioned at Fp1, I guess retina has received strong stimulation in both hippocampal and V4 experiments. Please discuss if this retinal stimulation, and not direct electrical stimulation of the brain, might have led to the observed results.**

We have investigated this issue in some detail in our previous paper, using recordings from the same hippocampal recording sites (Krause, Vieira, et al., 2019). First, we altered our stimulation montage so that retinal stimulation was held constant while the peak electric field was shifted far from our recording site. Entrainment disappeared, indicating that retinal activation could not account for the neural effects of tACS. Second, we showed that neural entrainment persists at frequencies above (40 Hz) and below (5 Hz) those that produce strong phosphenes in human subjects. Finally, during our hippocampal recordings, we made simultaneous recordings from TEO, a cortical visual area that is physically distant (~20mm) our hippocampal target but synaptically close to it. Effects arising from the retina would presumably propagate through the visual system before reaching the hippocampus, but we did not detect any increased entrainment in TEO neurons, even though hippocampal neurons were entrained.

These experiments are now summarized on page 7:

In Krause et al. (2019), we showed that a cortical visual area distant from the stimulation target was not entrained by tACS, suggesting that retinal input also does not produce the observed effects. Likewise, stimulation targeting the contralateral hemisphere, but producing similar retinal and somatosensory input, failed to entrain neurons at our recording site.

The phase of entrainment (discussed below) also argues against a retinal origin for this entrainment. Retinal input would have to pass through multiple synapses in the LGN, V1, and V2 before arriving at our V4 recording site; even more synapses lie between the retina and hippocampus, which should introduce a considerable phase lag.

**4)      At the end of the results section, there is an interesting part about the effective phase of tACS. The authors claim that a direct stimulation would result in spikes mainly locked to 90 degrees of the tACS current. What is the logic behind that? As neural membrane integrates the stimulation current, I assume that this phase is dependent on the stimulation current, and increasing the tACS current would decrease this phase towards zero. If this be current, then it would be hard to make conclusions using the preferred tACS phase. Please discuss.**

This logic is similar to that described by Filmer, Mattingley, and Dux (2020), which we now reference in the manuscript. Since the tACS waveform is sinusoidal, depolarizing current flows during the one half of the cycle, while hyperpolarizing current flows in the other, peaking at 90˚ and 270˚. Since firing is preferentially facilitated during depolarization and hindered during hyperpolarization, we reasoned that a direct effect would tend to concentrate spiking activity near the peak at 90˚, just as Figure S2 shows.

The Reviewer is correct that only a certain quantum of charge is needed to elicit an action potential and that sufficiently strong stimulation might deliver that amount even before reaching the peak. However, as the Reviewer mention above in Points #1 and #2, the field strengths produced during primate tACS are comparatively weak. An electric field of 1 V/m polarizes neurons by 0.2--0.5 mV, depending on their shape and orientation relative to the field (Deans, Powell, & Jefferys, 2007; Radman et al., 2009). This is sufficient to modulate responses to the cells’ other synaptic input, but is 20-100x too weak to directly elicit action potentials from quiescent neurons (Radman et al., 2009). Any phase precession would therefore be small and, if it did occur, could only occur in one direction: spikes might occur before the peak, but are unlikely to occur much after it, and are even less likely to occur during the hyperpolarizing part of the cycle.

The stimulation of somatosensory afferents peaks at the same time. To produce the entrainment we observed, that input would then need to travel down to the pons, ascend through the somatosensory system, and then somehow reach our recording sites in the hippocampus and V4. Each of these stages imposes conduction and synaptic transmission delays that would shift the phase of entrainment later in time. The stronger electric fields in the skin could counteract this by causing neurons to fire earlier in the tACS cycle, but it would be a remarkable coincidence if these factors exactly cancelled each other so as to be indistinguishable from a direct effect. This coincidence can also only occur for a single tACS frequency, as the synaptic and conduction delays are fixed by the anatomical properties of the pathway, but the time to peak changes with stimulation frequency. However, our previous study (without topical anesthesia) found that tACS shifted neurons’ firing phase to 90˚ at stimulation frequencies across three octaves (5-40 Hz).

We have revised our description of this analysis on p. 7 to make the logic more clear:

Examining the stimulation phase at which neurons become entrained provides an alternative method for distinguishing between direct and indirect effects (Filmer, Mattingley, & Dux, 2020). During tACS, the preferred firing phases of individual neurons are significantly concentrated around 90˚ (*V-*test: p = 0.0121 for **Θ_0_** = 90˚), an effect that is not present during sham (p = 0.75). Since the electric field’s depolarizing influence is strongest in both the skin and brain at 90˚, where the tACS waveform peaks, this is consistent with a direct effect on neurons within the stimulated area. In contrast, indirect peripheral influences would likely arrive at our recording sites later, due to conduction and synaptic transmission delays. Only a handful of intervening synapses, each with a few millisecond delay (Harris & Stark, 1973; Katz & Miledi, 1965), would be sufficient to introduce a lag of 45˚ (6.25 ms) in the preferred phase. A very precise combination of lower firing thresholds in skin and specific conduction delays could reproduce this finding, but this remarkable coincidence could only occur at a specific tACS frequency, which is difficult to square with our previous results (Krause et al., 2019) demonstrating a shift towards 90˚ at 5-40 Hz.

**5)      From the text, I concluded that the tACS intensity is specified as the amplitude of the sine wave, and not the peak-to-peak. I suggest clearly mentioning this in the Transcranial Alternating Current Stimulation subsection of the methods part to prevent potential confusions.**

Thank you for the suggestion. Confusion about the difference lead to the correction mentioned in Point #1, so it is very important to be clear. The Methods (pg. 9, “Transcranial alternating current stimulation”) now reads:

For the neurophysiology experiments, current was linearly ramped up from 0 over 10 seconds, held at full amplitude (±1 or ±2 mA; i.e., 2 or 4 mA peak-to-peak) for five minutes, and then ramped back down to 0, again over 10 seconds…

The ± symbol has also been added to all mentions of stimulation intensity to reinforce this point.

**6)      I could not find how the ceiling in Figure 2 is defined. Moreover, as a t-test compares the mean of the population to a hypothetical mean, I could not understand how the t-test for that is applied. Please clarify and mention the details in the manuscript. Please also clarify what each gray dot represents. Does each of them represent the fixation duration for a single trial or some statistical feature of several trials? Please clarify this in the manuscript.**

Ceiling was defined 100% time-on-task, which is now indicated on page 4. We agree with the reviewer that hypothesis testing may not be an appropriate way to test whether our data reaches that ceiling. The sample mean (and median) of our data will be biased downwards because random variation can reduce the proportion of time on task, but can never increase it over 100%. We have therefore opted to replace the t-test against ceiling with a simple descriptive statement. Fig 1 and Fig 2 have been modified accordingly and the results section (p. 4) now says:

As Fig 2 shows, time-on-task was significantly increased (p < 0.05) at all stimulation amplitudes when tACS was applied to the anesthetized skin, as compared to control sites. Furthermore, median performance is near ceiling (100% time on task) for ±0.5 and ±1 mA stimulation on anesthetized skin, but below 80% for when stimulating at higher intensities or at the control sites. These data suggest that EMLA increases somatosensory thresholds to ~±1 mA, and reduces—but does not eliminate—sensation at ±2.0 mA, just as it does in humans (Asamoah, Khatoun, & Mc Laughlin, 2019).

Our description of the behavioral paradigm may have made the grey dots in Figure 2 hard to understand. In every experimental block, tACS was applied once to the control site (tACS only; yellow) and once to the anesthetized skin (tACS+T.A.; green), with the condition order re-randomized for each block. Grey points indicate the responses to an individual stimulation event, while dashed lines denote points from the same block; the difference between the tACS and tACS+T.A conditions is then analyzed via a Wilcoxon signed rank test.

**Reviewer #2: Vieira, Krause & Pack investigated the impact of cutaneous somatosensory co-stimulation as alternative explanation for the neuronal entrainment effects observed by tACS in monkeys. They used topical anesthesia (lidocaine) of the skin below the stimulation electrodes to reduce the sensory input originating from peripheral nerves locally innervating the skin, and found no significant reduction in entrainment of neuronal firing by topical anesthesia, while animals appeared less disturbed by the stimulation when topical anesthesia was applied. Given the ongoing discussion on the effectiveness of tACS in humans and the role of cutaneous peripheral co-stimulation effects (as raised by Asamoah et al. Nat Commun 2019), addressing these issues in non-human primates is particularly important and will be of great value to the wide community using non-invasive brain stimulation techniques in humans. This paper elegantly contributes to a better understanding of the severity of these co-stimulation effects. It is written in a balanced way also addressing the limitations of the presented data (even though I suggest to make some of these limitations even more clear, see comments below).**

**1.      In humans TACS sensations are indeed not necessarily prevented (but only reduced) for higher intensities. While lidocaine removes the sharpness of the sensation and the potential pain, rhythmic cutaneous sensations can still be perceived and reported. How is effective blocking of these rhythmic sensations (which could serve to entrain brain activity) guaranteed in monkeys that cannot even report whether or not the feel something? These painless sensations may not distract the subject but still cause peripheral entrainment.**

This is a fair point, and one that we have tried to acknowledge in the paper. Our argument is simply that, given the reduction in peripheral input attributable to lidocaine, one would expect that it would also decrease the entrainment of neurons through peripheral mechanisms. But we observe no reduction, despite having sufficient statistical power to detect one if it were there. While it is of course impossible to rule out subthreshold influences entirely, our results do indicate that somatosensory input is unlikely to be the sole factor, or even an important one. As the reviewer points out, this is important to our understanding of the mechanisms of tACS. We would summarize the evidence against a strong role for peripheral input as follows:

- Changing the site of *stimulation*, but holding the *recording* site constant has little effect on the overall level of peripheral input, but eliminates neural entrainment (Krause et al., 2019).
- Changing the site of *recording* but holding the *stimulation* sites constant has no effect on peripheral input, but again eliminates neural entrainment (Krause et al., 2019).
- Neural entrainment occurs at a phase that is not consistent with a role for peripheral input (this paper).
- Holding both the *stimulation* and *recording* sites constant, while manipulating peripheral input with topical anesthesia, does not affect neural entrainment (this paper).
- The behavioral stimulation thresholds reported in this paper are similar in humans and monkeys, suggesting that the lack of behavioral response in monkeys reflects a genuine lack of sensation, rather than an inability to express it.

These findings all converge on the same point: Peripheral sensation, if present, cannot account for the neural entrainment effects.

We now discuss this possibility on page 10:

Since our paradigm depends on tACS-induced sensations being surprising or noxious enough to distract the animal from the fixation task, we cannot completely exclude the possibility that some residual somatosensory input persists, despite the topical anesthesia. However, measurements from human subjects, who can provide richer reports of their subjective experiences, find very similar thresholds: ±0.51 mA under control conditions and ±0.93 mA after EMLA topical anesthesia (Asamoah, Khatoun, & Mc Laughlin, 2019), suggesting that our procedure accurately measures the monkey’s perceptual threshold.

The expected effects of topical anesthesia on supra-threshold stimulation are also unclear. In the HIGH condition of their Experiment 2A and 2B, Asamoah et al. (2019) applied currents of ±2.2 mA and ±4.7 mA to entrain physiological and pathological tremor, respectively. These are well above perceptual threshold (±0.93 mA, measured in the same subjects), and yet topical anesthesia reportedly prevented tremor entrainment entirely. These results seemed surprising to us, but it does seem plausible that, if somatosensory input were entraining neurons, reducing it would lead to a corresponding reduction in entrainment. We therefore tested both sub- and supra-threshold currents. We now discuss this rationale on page 4-5:

We therefore collected data using currents that were both at perceptual threshold (±1 mA; diamonds in Fig 3 and Fig S1) and above it (±2 mA; circles in Fig 3), reasoning that if somatosensory input were producing neuronal entrainment, reducing it either partially or completely would lead to a corresponding decrease or elimination of entrainment.

**2.      Additional recordings from primary somatosensory cortex (for the hippocampus or V4 targeting montage) would allow to assess the actual effectiveness of the blockade of cutaneous input to S1 during TACS and to compare it with and without topical anesthesia.**

We completely agree that recording neurons in somatosensory cortex would be interesting, but this is well beyond the scope of this paper. None of our animals’ implants provide access to somatosensory cortex, and obtaining/preparing a new monkey for recording would take 6-9 months and tens of thousands of dollars, even if our lab were not currently closed due to the ongoing pandemic.

**However, even then, the unintended direct peripheral co-stimulation of cranial nerves (e.g., trigeminal or vagal nerve) or the meninges (both not affected by the superficial topical anesthesia of the skin, but densely innervated) may affect a number of other brain regions (and their networks) without the entrainment necessarily being observable in S1. Especially two-electrode montages such as FP1 -O1/P7 likely target many peripheral neuronal structures that are not located in the skin itself (in humans FP1 is located very closely to the point where the first branch of the trigeminal nerve enters the skull through a small foramen funneling transcranial currents). In summary, an entrainment via peripheral nerve stimulation (not originating from the anesthetized past of the skin and thus equally strong with and without anesthesia) cannot be excluded. This should be made even more clear in the manuscript.**

This is an excellent point. There is a whole continuum of possible entrainment sources, with sensory stimulation at one end, direct polarization of central neurons on the other, and stimulation of cranial nerves somewhere in the middle. The data shown here strongly argues against the claim that somatosensory input is required for tACS to entrain neurons. As the Reviewer points out, the case against stimulation of cranial nerves is far less clear from our data.

We have revised the paper throughout to make it clearer that this paper focuses on the sensory end of that spectrum. The abstract now reads:

In particular, it has often been hypothesized that tACS acts on sensory fibers in the skin….

Blocking somatosensory input with a topical anesthetic does not significantly alter these neural entrainment effects. These data are therefore consistent with the direct stimulation hypothesis and suggest that peripheral somatosensory stimulation is not required for tACS to entrain neurons.

Likewise, the introduction on page says 3:

Rhythmic activation of somatosensory fibers could thus indirectly entrain central neurons by providing them with temporally structured sensory input. Since shunting also weakens electric fields in the brain, this *indirect* mechanism has been frequently proposed to be the dominant mode of action in humans….

An unambiguous test of the role of somatosensory input is to directly measure neural entrainment during tACS, with and without topical anesthetic. Here, we perform that decisive experiment in non-human primates….

In addition, we now discuss the possible role of cranial nerves in the Discussion on page 8:

In these experiments, we applied topical anesthesia to a 5 cm (diameter) circle of skin under and around each electrode. Since skin is highly conductive, current could spread to peripheral nerve fibers outside that area, albeit not to an extent that affects behavior (Figure 2). Stimulation of larger fibers, including cranial nerves, may be a particular concern, as they are deeper in the skin and therefore less affected by topical anesthesia. In fact, this mechanism may account for some of the reported somatosensory effects. In (Asamoah, Khatoun, & Mc Laughlin, 2019), the control stimulation that was applied to the forelimb skin may also drive the medial or radial nerves (Li et al., 2013); stimulation of these fibers has been reported to reduce tremor in humans (Lin et al., 2018). That said, there are reasons to believe that cranial nerve stimulation may not be a major factor in our data. To the extent that the high conductivity of skin allows distant cranial nerves to be stimulated, it should also make any resulting effects relatively insensitive to the position of the electrodes. However, the effects of tACS appear to depend on the precise locations of the stimulating electrode. In our previous study, shifting electrodes onto the contralateral hemisphere (~3-4 cm) was sufficient completely abolish entrainment, even though electric field in the skin was similar in both cases. A number of experiments have also demonstrated that specific electrode montages are required to produce the hypothesized behavioral effects (Khatoun et al., 2018; Mehta et al., 2015). Peripheral and central stimulation also appear to have synergistic effects, suggesting the existence of separate mechanisms (Celnik et al., 2009). Finally, as with somatosensory input, it remains unclear how stimulation of the cranial nerves would alter the timing of spiking activity in synaptically distant area V4 or the hippocampus. However, given the diverse phenomena associated with vagal nerve activity (Zanos, 2019), this possibility cannot be discounted altogether.

**3.      The authors used specific montages for stimulating hippocampus or V4, but not the one that was used previously to show the effect on (physiological) tremor entrainment (Asamoah et al.). Maybe the precise montage location (relative to cranial nerves etc.) is indeed crucial for whether or not a strong peripheral co-stimulation mediated entrainment can be observed. So the conclusion of tACS with and without topical anesthesia being equally effective in entraining neurons in the brain has to be limited to the two tested montages.**

We agree that our conclusions are in no way definitive for the entire brain, and now acknowledge the myriad possible sources of entrainment in the Discussion on page 8:

The combined effects of sensory input, nerve fiber stimulation, and direct polarization will necessarily vary across stimulation protocols, targeted brain regions, and behavioral tasks, frustrating any simplistic attempt at interpretation.

However, the fact that external electric fields polarize neurons is well established from fundamental biophysical principles and *in vitro* experiments where most possible confounds (e.g., somatosensation) are not present. As far as we know, there is no reason to suspect that this same mechanism would not be operative throughout the brain as well, anywhere it is possible to generate electric fields of sufficient strength. It is certainly possible that other mechanisms could co-exist with, or even dominate, the effects of direct membrane polarization. However, those mechanisms would seem to require very specific and as-of-yet hypothetical patterns of connectivity, and should therefore be considered on a case-by-case basis instead.

**Reviewer #3: This is an interesting and timely report and the authors provide compelling evidence proving the direct effect of tACS upon cortical neurons. The results clearly indicate that even when somatosensory input is blocked by EMLA cream, 20 Hz tACS was still entraining neural activity, thus providing experimental evidence that tACS reached deep cortical structures such as the hippocampus as well as in more superficial visual area V4. Conclusions are fully supported by the data. The method is sound and the results are clear.**

Thank you for the very supportive comments!

**I only have a minor comment/question, that is why they only used 20 Hz stimulation to entrain neurons. They refer to their previous work, but my understanding is that 20 Hz might have differential effects compared to 10 Hz, and their effects are often compared in studies with human participants. While the specific effect of frequency of stimulation is not central to the purpose of the study, I was wondering whether different frequencies might affect cortical neurons and/or indirect somatosensory responses differently.**

We have added a paragraph explaining why we focused on a single stimulation frequency to the “Transcranial Alternating Current Stimulation” section of the Methods, on page 9:

Stimulation consisted of a 20 Hz sinusoidal waveform. In our previous experiments (Krause et al., 2019), we found that tACS at this frequency entrained about half of the hippocampal neurons we recorded; similar results were also obtained with 5, 10, and 40 Hz stimulation. Since human subjects also report similar sensations when receiving tACS at frequencies between 2-64 Hz (Raco et al., 2014), the neural and somatosensory aspects of our study should generalize to the tACS protocols used in most human experiments.

The Reviewer is correct that many tACS experiments use 10 Hz stimulation, but 20 Hz tACS has also been used in a number of studies exploring the role of beta oscillations, including:

- Wischnewski, Schutter, and Nitsche (2019)
- Wiener et al. (2018)
- Feurra et al. (2011)
- Naros and Gharabaghi (2017)

The “right” frequency likely depends on many factors, including the targeted brain region, the behavior or phenomenon of interest, and potentially even the individual subject.

# References

Alekseichuk, I., Mantell, K., Shirinpour, S., & Opitz, A. (2019). Comparative modeling of transcranial magnetic and electric stimulation in mouse, monkey, and human. *Neuroimage, 194*, 136-148. doi:10.1016/j.neuroimage.2019.03.044

Asamoah, B., Khatoun, A., & Mc Laughlin, M. (2019). tACS motor system effects can be caused by transcutaneous stimulation of peripheral nerves. *Nat Commun, 10*(1), 266. doi:10.1038/s41467-018-08183-w

Bahmani, Z., Daliri, M. R., Merrikhi, Y., Clark, K., & Noudoost, B. (2018). Working Memory Enhances Cortical Representations via Spatially Specific Coordination of Spike Times. *Neuron, 97*(4), 967-979 e966. doi:10.1016/j.neuron.2018.01.012

Celnik, P., Paik, N. J., Vandermeeren, Y., Dimyan, M., & Cohen, L. G. (2009). Effects of combined peripheral nerve stimulation and brain polarization on performance of a motor sequence task after chronic stroke. *Stroke, 40*(5), 1764-1771. doi:10.1161/STROKEAHA.108.540500

Datta, A., Bansal, V., Diaz, J., Patel, J., Reato, D., & Bikson, M. (2009). Gyri-precise head model of transcranial direct current stimulation: improved spatial focality using a ring electrode versus conventional rectangular pad. *Brain Stimul, 2*(4), 201-207, 207 e201. doi:10.1016/j.brs.2009.03.005

Deans, J. K., Powell, A. D., & Jefferys, J. G. (2007). Sensitivity of coherent oscillations in rat hippocampus to AC electric fields. *J Physiol, 583*(Pt 2), 555-565. doi:10.1113/jphysiol.2007.137711

Feurra, M., Bianco, G., Santarnecchi, E., Del Testa, M., Rossi, A., & Rossi, S. (2011). Frequency-dependent tuning of the human motor system induced by transcranial oscillatory potentials. *J Neurosci, 31*(34), 12165-12170. doi:10.1523/JNEUROSCI.0978-11.2011

Filmer, H. L., Mattingley, J. B., & Dux, P. E. (2020). Modulating brain activity and behaviour with tDCS: Rumours of its death have been greatly exaggerated. *Cortex, 123*, 141-151. doi:10.1016/j.cortex.2019.10.006

Foster, B. L., Kaveh, A., Dastjerdi, M., Miller, K. J., & Parvizi, J. (2013). Human retrosplenial cortex displays transient theta phase locking with medial temporal cortex prior to activation during autobiographical memory retrieval. *J Neurosci, 33*(25), 10439-10446. doi:10.1523/JNEUROSCI.0513-13.2013

Harris, D. A., & Stark, L. (1973). Synaptic delay: its effect on information transmission in the crayfish caudal photoreceptor system. *Brain Res, 51*, 340-344. doi:10.1016/0006-8993(73)90386-7

Huang, Y., Liu, A. A., Lafon, B., Friedman, D., Dayan, M., Wang, X., . . . Parra, L. C. (2017). Measurements and models of electric fields in the in vivo human brain during transcranial electric stimulation. *Elife, 6*. doi:10.7554/eLife.18834

Jackson, M. P., Rahman, A., Lafon, B., Kronberg, G., Ling, D., Parra, L. C., & Bikson, M. (2016). Animal models of transcranial direct current stimulation: Methods and mechanisms. *Clin Neurophysiol, 127*(11), 3425-3454. doi:10.1016/j.clinph.2016.08.016

Johnson, L., Alekseichuk, I., Krieg, J., Doyle, A., Yu, Y., Vitek, J., . . . Opitz, A. (2019). Dose-Dependent Effects of Transcranial Alternating Current Stimulation on Spike Timing in Awake Nonhuman Primates. *bioRxiv*, 696344. doi:10.1101/696344

Katz, B., & Miledi, R. (1965). The Measurement of Synaptic Delay, and the Time Course of Acetylcholine Release at the Neuromuscular Junction. *Proc R Soc Lond B Biol Sci, 161*, 483-495. doi:10.1098/rspb.1965.0016

Khatoun, A., Breukers, J., Op de Beeck, S., Nica, I. G., Aerts, J. M., Seynaeve, L., . . . Mc Laughlin, M. (2018). Using high-amplitude and focused transcranial alternating current stimulation to entrain physiological tremor. *Sci Rep, 8*(1), 4927. doi:10.1038/s41598-018-23290-w

Krause, M. R., Vieira, P. G., Csorba, B. A., Pilly, P. K., & Pack, C. C. (2019). Transcranial alternating current stimulation entrains single-neuron activity in the primate brain. *Proc Natl Acad Sci U S A, 116*(12), 5747-5755. doi:10.1073/pnas.1815958116

Li, R., Hettinger, P. C., Machol, J. A., Liu, X., Stephenson, J. B., Pawela, C. P., . . . Hyde, J. S. (2013). Cortical plasticity induced by different degrees of peripheral nerve injuries: a rat functional magnetic resonance imaging study under 9.4 Tesla. *J Brachial Plex Peripher Nerve Inj, 8*(1), 4. doi:10.1186/1749-7221-8-4

Lin, P. T., Ross, E. K., Chidester, P., Rosenbluth, K. H., Hamner, S. R., Wong, S. H., . . . Delp, S. L. (2018). Noninvasive neuromodulation in essential tremor demonstrates relief in a sham-controlled pilot trial. *Mov Disord, 33*(7), 1182-1183. doi:10.1002/mds.27350

Mehta, A. R., Pogosyan, A., Brown, P., & Brittain, J. S. (2015). Montage matters: the influence of transcranial alternating current stimulation on human physiological tremor. *Brain Stimul, 8*(2), 260-268. doi:10.1016/j.brs.2014.11.003

Miranda, P. C., Lomarev, M., & Hallett, M. (2006). Modeling the current distribution during transcranial direct current stimulation. *Clin Neurophysiol, 117*(7), 1623-1629. doi:10.1016/j.clinph.2006.04.009

Naros, G., & Gharabaghi, A. (2017). Physiological and behavioral effects of beta-tACS on brain self-regulation in chronic stroke. *Brain Stimul, 10*(2), 251-259. doi:10.1016/j.brs.2016.11.003

Raco, V., Bauer, R., Olenik, M., Brkic, D., & Gharabaghi, A. (2014). Neurosensory effects of transcranial alternating current stimulation. *Brain Stimul, 7*(6), 823-831. doi:10.1016/j.brs.2014.08.005

Radman, T., Ramos, R. L., Brumberg, J. C., & Bikson, M. (2009). Role of cortical cell type and morphology in subthreshold and suprathreshold uniform electric field stimulation in vitro. *Brain Stimul, 2*(4), 215-228, 228 e211-213. doi:10.1016/j.brs.2009.03.007

Rutishauser, U., Ross, I. B., Mamelak, A. N., & Schuman, E. M. (2010). Human memory strength is predicted by theta-frequency phase-locking of single neurons. *Nature, 464*(7290), 903-907. doi:10.1038/nature08860

Sadleir, R. J., Vannorsdall, T. D., Schretlen, D. J., & Gordon, B. (2010). Transcranial direct current stimulation (tDCS) in a realistic head model. *Neuroimage, 51*(4), 1310-1318. doi:10.1016/j.neuroimage.2010.03.052

Spencer, K. M., Nestor, P. G., Perlmutter, R., Niznikiewicz, M. A., Klump, M. C., Frumin, M., . . . McCarley, R. W. (2004). Neural synchrony indexes disordered perception and cognition in schizophrenia. *Proc Natl Acad Sci U S A, 101*(49), 17288-17293. doi:10.1073/pnas.0406074101

van Wingerden, M., Vinck, M., Lankelma, J., & Pennartz, C. M. (2010a). Theta-band phase locking of orbitofrontal neurons during reward expectancy. *J Neurosci, 30*(20), 7078-7087. doi:10.1523/JNEUROSCI.3860-09.2010

van Wingerden, M., Vinck, M., Lankelma, J. V., & Pennartz, C. M. (2010b). Learning-associated gamma-band phase-locking of action-outcome selective neurons in orbitofrontal cortex. *J Neurosci, 30*(30), 10025-10038. doi:10.1523/JNEUROSCI.0222-10.2010

Vinck, M., Battaglia, F. P., Womelsdorf, T., & Pennartz, C. (2012). Improved measures of phase-coupling between spikes and the Local Field Potential. *J Comput Neurosci, 33*(1), 53-75. doi:10.1007/s10827-011-0374-4

Vinck, M., Bos, J. J., Van Mourik-Donga, L. A., Oplaat, K. T., Klein, G. A., Jackson, J. C., . . . Pennartz, C. M. (2015). Cell-Type and State-Dependent Synchronization among Rodent Somatosensory, Visual, Perirhinal Cortex, and Hippocampus CA1. *Front Syst Neurosci, 9*, 187. doi:10.3389/fnsys.2015.00187

Vinck, M., Lima, B., Womelsdorf, T., Oostenveld, R., Singer, W., Neuenschwander, S., & Fries, P. (2010). Gamma-phase shifting in awake monkey visual cortex. *J Neurosci, 30*(4), 1250-1257. doi:10.1523/JNEUROSCI.1623-09.2010

Voroslakos, M., Takeuchi, Y., Brinyiczki, K., Zombori, T., Oliva, A., Fernandez-Ruiz, A., . . . Berenyi, A. (2018). Direct effects of transcranial electric stimulation on brain circuits in rats and humans. *Nat Commun, 9*(1), 483. doi:10.1038/s41467-018-02928-3

Wiener, M., Parikh, A., Krakow, A., & Coslett, H. B. (2018). An Intrinsic Role of Beta Oscillations in Memory for Time Estimation. *Sci Rep, 8*(1), 7992. doi:10.1038/s41598-018-26385-6

Wischnewski, M., Schutter, D., & Nitsche, M. A. (2019). Effects of beta-tACS on corticospinal excitability: A meta-analysis. *Brain Stimul, 12*(6), 1381-1389. doi:10.1016/j.brs.2019.07.023

Zanos, T. P. (2019). Recording and Decoding of Vagal Neural Signals Related to Changes in Physiological Parameters and Biomarkers of Disease. *Cold Spring Harb Perspect Med, 9*(12). doi:10.1101/cshperspect.a034157
